# Multinomial logistic regression algorithm for the classification of patients with parkinsonisms

**DOI:** 10.1186/s13550-025-01210-0

**Published:** 2025-03-16

**Authors:** Eva Štokelj, Tomaž Rus, Jan Jamšek, Maja Trošt, Urban Simončič

**Affiliations:** 1 Faculty of Mathematics and Physics, University of Ljubljana, Jadranska ulica 19, 1000 Ljubljana, Slovenia; 2 Department of Neurology, University Medical Centre Ljubljana, Zaloška cesta 2, 1000 Ljubljana, Slovenia; 3 Department of Nuclear Medicine, University Medical Centre Ljubljana, Zaloška cesta 7, 1000 Ljubljana, Slovenia; 4 Faculty of Medicine, University of Ljubljana, Vrazov trg 2, 1000 Ljubljana, Slovenia; 5 Jožef Stefan Institute, Jamova cesta 39, 1000 Ljubljana, Slovenia

**Keywords:** Parkinsonisms, Metabolic brain pattern, Multinomial logistic regression, FDG-PET, SSM/PCA

## Abstract

**Background:**

Accurate differential diagnosis of neurodegenerative parkinsonisms is challenging due to overlapping early symptoms and high rates of misdiagnosis. To improve the diagnostic accuracy, we developed an integrated classification algorithm using multinomial logistic regression and Scaled Subprofile Model/Principal Component Analysis (SSM/PCA) applied to ^18^F-fluorodeoxyglucose positron emission tomography (FDG-PET) brain images. In this novel classification approach, SSM/PCA is applied to FDG-PET brain images of patients with various parkinsonisms, which are compared against the constructed undetermined images. This process involves spatial normalization of the images and dimensionality reduction via PCA. The resulting principal components are then used in a multinomial logistic regression model, which generates disease-specific topographies that can be used to classify new patients. The algorithm was trained and optimized on a cohort of patients with neurodegenerative parkinsonisms and subsequently validated on a separate cohort of patients with parkinsonisms.

**Results:**

The Area Under the Curve (AUC) values were the highest for progressive supranuclear palsy (PSP) (AUC = 0.95), followed by Parkinson’s disease (PD) (AUC = 0.93) and multiple system atrophy (MSA) (AUC = 0.90). When classifying the patients based on their calculated probability for each group, the desired tradeoff between sensitivity and specificity had to be selected. With a 99% probability threshold for classification into a disease group, 82% of PD patients, 29% of MSA patients, and 77% of PSP patients were correctly identified. Only 5% of PD, 6% of MSA and 6% of PSP patients were misclassified, whereas the remaining patients (13% of PD, 65% of MSA and 18% of PSP) are undetermined by our classification algorithm.

**Conclusions:**

Compared to existing algorithms, this approach offers comparable accuracy and reliability in diagnosing PD, MSA, and PSP with no need of healthy control images. It can also distinguish between multiple types of parkinsonisms simultaneously and offers the flexibility to easily accommodate new groups.

**Supplementary Information:**

The online version contains supplementary material available at 10.1186/s13550-025-01210-0.

## Introduction

Neurodegenerative parkinsonisms are syndromes that present with bradykinesia, rigidity and tremor and predominantly affect elderly population [1, 2]. Among them, the most common is idiopathic Parkinson’s disease (PD), while atypical parkinsonisms such as multiple system atrophy (MSA), progressive supranuclear palsy (PSP), and corticobasal degeneration (CBD) [3] are rare. Despite sharing similar early symptoms, these disorders vary in some clinical characteristics, progression rates and prognosis. Accurate early diagnosis is crucial for effective patient management, enrolment in clinical trials, and better understanding of the pathophysiology of these syndromes. The diagnosis of parkinsonisms is clinical, but may be wrong in up to 25% of cases [4, 5].

Molecular imaging methods have emerged as valuable tools for increasing diagnostic accuracy in these syndromes. While all neurodegenerative parkinsonisms exhibit presynaptic dopaminergic pathway dysfunction, there are some subtle differences among them in radiopharmaceutical distribution through the striatum but they are generally insufficient to reliably differentiate them on individual subject level [6, 7]. Postsynaptic D2 receptor imaging can help distinguish PD from atypical parkinsonisms, but its overall accuracy remains relatively low [8]. Similarly, imaging postganglionic sympathetic cardiac innervation with ^123^I-metaiodobenzylguanidine (MIBG) can differentiate PD from MSA, yet it also shows limited specificity [9, 10]. In recent years, radiopharmaceuticals that bind pathological tau proteins have shown promise in differentiating tauopathies (PSP and CBD) from other parkinsonian disorders, and preliminary research on synuclein imaging indicates a potential role in diagnosing synucleinopathies such as PD and MSA [11, 12]. Nonetheless, due to complex biochemistry, intracellular accumulation in diverse cell types, and typically low or variable target protein levels in the brain, these methods are not yet feasible for routine clinical use.

Currently, the most powerful and widely used diagnostic approach for differentiating among neurodegenerative parkinsonian syndromes is molecular imaging of brain metabolism, particularly with ^18^F-fluorodeoxyglucose positron emission tomography (FDG-PET) [13, 14]. FDG-PET can detect metabolic changes that often precede clinical symptoms and structural abnormalities, making it the recommended modality for differentiating parkinsonisms in clinical practice [15]. However, visual interpretation of FDG-PET requires significant reader expertise.

To address this, several mathematical and statistical approaches have been employed to enhance the diagnostic capability of FDG-PET [16]. One of the most commonly used is the scaled subprofile model (SSM) based on principal component analysis (PCA) and binomial logistic regression [17]. This method applied to FDG-PET images has been utilized to identify a parkinsonism specific metabolic brain patterns. The expression of these patterns enables us to discriminate between parkinsonian patients and healthy controls (HC) [17]. These patterns include PD-related patterns (PDRP), MSA-related patterns (MSARP), PSP-related patterns (PSPRP) and CBD-related pattern (CBDRP). They have been identified and validated in various cohorts [18–27]. Additionally, a multi-level logistic regression [28–30] and several other machine learning algorithms [31–33] have been developed to improve the differential diagnosis of parkinsonisms [34]. However, previous differential diagnostic approaches often involved numerous analytical steps, increasing the risk of error accumulation [28–30] or were unsupervised, lacking information on features driving classification [33]. Therefore, there was a clear imperative to develop new methodological approaches to address these shortcomings.

Our study aimed to develop and apply an integrated single level classification algorithm based on multinomial logistic regression and Scaled Subprofile Model/Principal Component Analysis (SSM/PCA), which can simultaneously distinguish between different parkinsonisms and provide a characteristic disease-related metabolic topographies that distinguish among PD, MSA and PSP.

## Subjects and methods

### Subjects

A total of 161 neurodegenerative parkinsonian patients were enrolled, comprising of 87 patients with PD, 37 with MSA, and 37 with PSP, for the application of the multinomial logistic regression algorithm. We focused on the three most common parkinsonian syndromes for this initial study, with the possibility of expanding to other disorders in future research. Only patients without pronounced vascular or other structural lesions, considerable brain atrophy, history of head trauma or encephalitis were enrolled. We selected patients with Parkinson’s disease in their middle stage of the disease and patients with MSA in PSP in more advanced stage, to be confident about their clinical diagnosis.

The patients were divided into two cohorts: Cohort A, consisting of 60 patients (20 from each of the PD, MSA, and PSP groups), was used for the model identification. The remaining patients were assigned to Cohort B for the validation purposes. All individuals included in the study were clinically diagnosed by a movement disorders specialist following current clinical criteria [2, 35, 36] and were subsequently followed up for at least one year after the FDG-PET scan to confirm the diagnosis at the Movement Disorders Clinic at the University Medical Centre Ljubljana (UMCL). They underwent brain FDG-PET imaging at the Department for Nuclear Medicine at UMCL as part of previous studies [18, 19, 30]. Subjects’ demographic characteristics are presented in Table 1. The study was approved by the National Medical Ethics Committee of the Republic of Slovenia. All participants had to sign an informed consent waiver before they were injected with the radiotracer, where they agreed to participate in the imaging part of the study and provide their imaging data for further statistical analysis.

**Table 1:** Demographic data

	Cohort A			Cohort B		
	PD	MSA	PSP	PD	MSA	PSP
N	20	20	20	67	17	17
Gender (M/F)	16/4	9/11	14/6	38/29**	7/10	7/10**
Age (yrs)*	70.1 ± 7.8	63.0 ± 9.7	70.0 ± 8.3	68.8 ± 9.5	67.4 ± 9.4	75.2 ± 4.8
Disease duration (yrs)*	4.3 ± 4.1	5.0 ± 2.9	5.0 ± 1.6	5.1 ± 3.5	4.2 ± 2.6	4.8 ± 3.0

Cohort A: FDG-PET scans used for the model identification. Cohort B: FDG-PET scans used for model validation

*Age and disease duration are given as mean ± standard deviation

**Significantly different from Cohort A at *p* < 0.01

FDG-PET imaging was performed on a Siemens Biograph mCT PET/CT scanner 35–45 min after the injection of 250 MBq of FDG. The DICOM images obtained from the FDG-PET scanner underwent preprocessing, which involved spatial normalization into a standard Montreal Neurological Institute (MNI)-based PET template and smoothing with a Gaussian kernel of 10 × 10 × 10 mm FWHM. The resulting image resolution was approximately 12 mm FWHM, compliant with guidelines on image resolution [37]. Preprocessing was performed using the Statistical Parametric Mapping 5 (SPM5) software (Institute of Neurology, UCL, London, UK), running on Matlab 7.0 (MathWorks Inc., Natick, MA). More detailed information can be found in protocol description outlined in previous studies [18, 19].

### Model identification

**Fig. 1:**
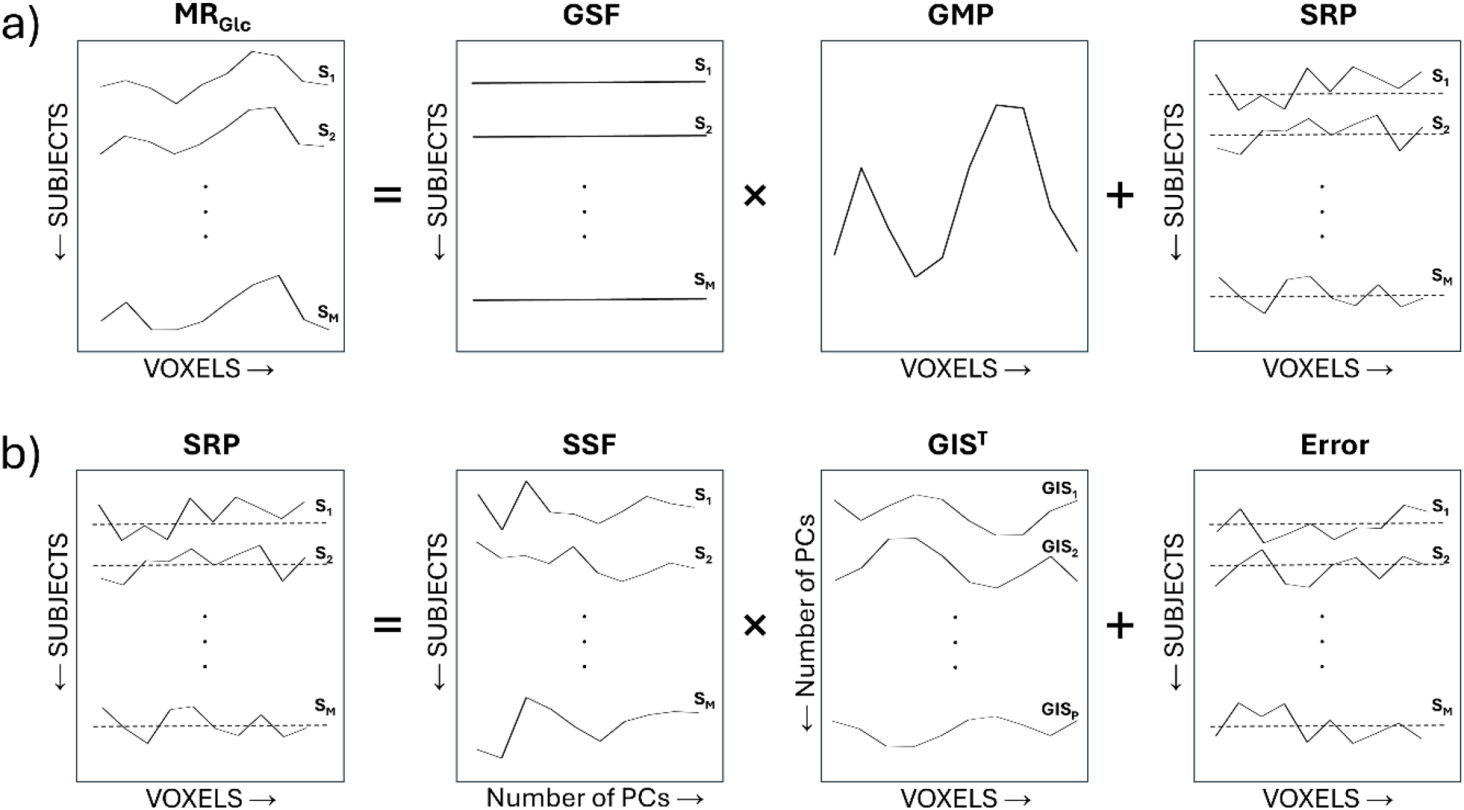
Schematic description of the scaled subprofile model/principal component analysis (SSM/PCA) method. **a**) A SSM decomposition of subjects’ profiles of metabolic rate of glucose (MRGlc) into two parts: a group mean profile (GMP), individually scaled with global scaling factor (GSF) for each subject, and the remaining subject residual profile (SRP). **b**) The SRP can be further decomposed into two parts: the subject scaling factors (SSF) multiplied by the group invariant subprofile (GIS) and the error

The SSM/PCA algorithm is a statistical method applied to brain imaging to analyze variance and covariance in the images, and to identify a significant group-dependent and region-specific effects [38]. It builds on the assumption that the individual PET image can be decomposed into two parts: the individually scaled group mean profile and the subject’s specific residual profile, as sketched in Fig. 1a. The SSM/PCA analyze the subject’s specific residual profiles (Fig. 1b) that is obtained by double-cantering the log-transformed subjects’ profiles of metabolic rate of glucose.

**Fig. 2:**
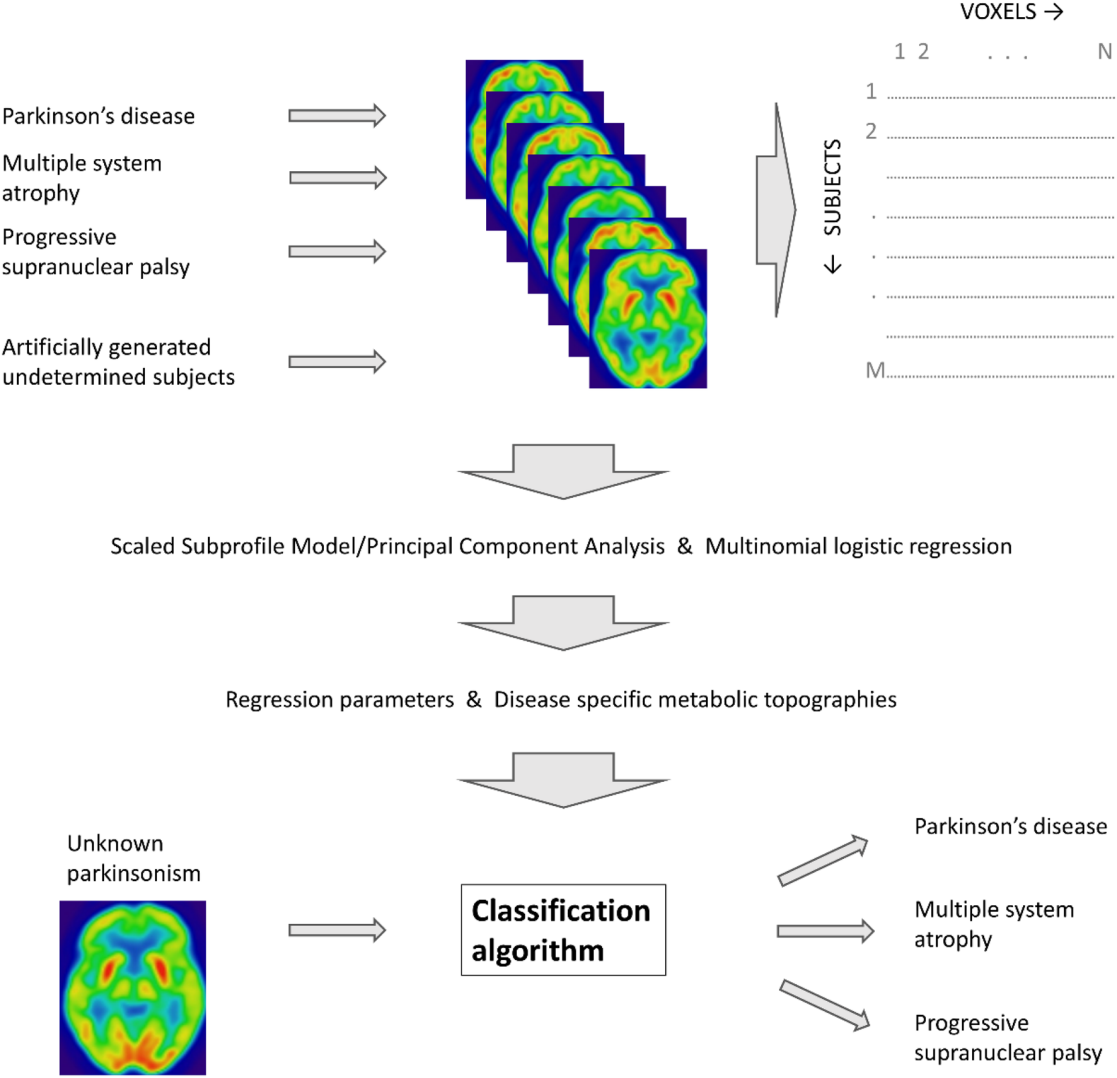
A novel algorithm using FDG-PET images and multinomial logistic regression is based on Scaled Subprofile Model/Principal Component Analysis. It distinguishes between Parkinson’s disease, multiple system atrophy, and progressive supranuclear palsy. The algorithm involves spatial normalization of the images and dimensionality reduction via PCA. The resulting principal components are then used in a multinomial logistic regression model, which generates disease-specific topographies that can be used to classify new patients

It is commonly employed for classification, traditionally in binary settings. Unlike binary classification, we analyzed more than two groups of patients: PD, MSA, PSP, and the reference group of undetermined subjects (UD). Although each of these disorders has subtypes (e.g., cerebellar type MSA, parkinsonian type MSA, Richardson subtype PSP, parkinsonian subtype PSP, …), several previous research has demonstrated that the subtypes exhibit common metabolic network changes and were therefore considered a single entity for the purpose of analysis [19, 39]. Each UD image represented the average of one PD, one MSA, and one PSP image from the cohort A. They served as a reference for the multinomial logistic regression analysis. Importantly, each PD, MSA, or PSP image contributed only once to the creation of UD images to ensure their independence. We used the average images because we wanted to create an algorithm that does not require healthy controls, which may be the issue for many centers. Instead of healthy controls we wanted to use images, whose patterns resemble some patterns of each group (therefore undetermined). One possible mathematical transformation to this goal is to take average of three images, each with one syndrome. The algorithm is outlined in Fig. 2. Within this framework, we derived three final topographies (i.e. disease specific combination of principal components). Each topography delineated the metabolic spatial profile of one syndrome in comparison to the UD reference group.

In the first step, 60 FDG-PET brain images from the Cohort A were utilized. Additionally, 20 new UD images were created, so altogether 80 FDG-PET images were analyzed. These 80 FDG-PET images were transformed into a single row vector, masked with threshold (varied from 15 to 45% of the FDG-PET maximum value) and voxels inside the mask were assembled into a matrix **P** of size (M,N). Here, M denotes the number of subjects and N signifies the number of voxels included in the SSM/PCA. The matrix **P** undergoes a logarithmic transformation, which converts multiplicative effects into additive ones. These additive effects are then eliminated through a process called double centering. The mean value is extracted from each row (representing different subjects), which forms a new matrix **Q**. A similar process is carried out for the columns (representing different voxels), so we get the Subject Residual Profile (${\mathbf{SRP}}$) matrix. The ${\mathbf{SRP}}$ matrix represents the differences between all subjects and all voxels in the identification group.

1 $${\mathbf{Q}}_{{{\varvec{ij}}}} = {\text{log }}{\mathbf{P}}_{{{\varvec{ij}}}} - \frac{1}{{\text{N}}}\mathop \sum \limits_{{\varvec{j}}} {\text{log }}{\mathbf{P}}_{{{\varvec{ij}}}} ,$$

2 $${\mathbf{SRP}}_{{{\varvec{ij}}}} = {\mathbf{Q}}_{{{\varvec{ij}}}} - \frac{1}{{\text{M}}}\mathop \sum \limits_{{\varvec{i}}} {\mathbf{Q}}_{{{\varvec{ij}}}} .$$

From the ${\mathbf{SRP}}$, we calculate the subject covariance matrix:

3 $${\mathbf{S}}_{{{\mathbf{sub}}}} = {\mathbf{SRP}} \cdot {\mathbf{SRP}}^{{\mathbf{T}}} .$$

Then we calculate its eigenvalues, Λ, and eigenvectors, **E**, using the equation:

4 $${\mathbf{S}}_{{{\mathbf{sub}}}} \cdot {\mathbf{E}} = {\mathbf{E}} \cdot {\Lambda }{.}$$

Same eigenvalues Λ are also eigenvalues of the voxel covariance matrix ${\mathbf{S}}_{{{\mathbf{vox}}}} = {\mathbf{SRP}}^{{\mathbf{T}}} \cdot {\mathbf{SRP}}$:

5 $${\mathbf{S}}_{{{\mathbf{vox}}}} \cdot {\mathbf{SRP}}^{{\mathbf{T}}} \cdot {\mathbf{E}} = {\mathbf{SRP}}^{{\mathbf{T}}} \cdot {\mathbf{E}} \cdot {\Lambda }.$$

Matrix ${\mathbf{SRP}}^{{\mathbf{T}}} \cdot {\mathbf{E}}$ is matrix of eigenvectors for the voxel covariance matrix ${\mathbf{S}}_{{{\mathbf{vox}}}}$. Those eigenvectors are then used to define the Group Invariant Subprofiles (${\mathbf{GIS}}$) as:

6 $${\mathbf{GIS}} = {\mathbf{SRP}}^{{\varvec{T}}} \cdot {\mathbf{E}} \cdot {\Lambda }^{{ - \frac{1}{2}}} .$$

They represent the orthonormal base for the ${\mathbf{SRP}}$. Therefore, the ${\mathbf{SRP}}$ can be expressed in terms of this orthonormal base as:

7 $${\mathbf{SRP}} = {\mathbf{SSF}} \cdot {\mathbf{GIS}}^{{\varvec{T}}} .$$

The subject scores field (${\mathbf{SSF}}$) can be evaluated with the eigenvalues, Λ, and eigenvectors, **E**, as:

8 $${\mathbf{SSF}} = {\mathbf{E}} \cdot {\Lambda }^{\frac{1}{2}}$$

Now we recall the Eq. 7 that express the ${\mathbf{SRP}}$ in terms of the orthonormal base ${\mathbf{GIS}}$. We recognize that some of ${\mathbf{GIS}}$ eigenvectors represent differences among the patients that are not related to syndromes. Therefore, we consider only a certain number of these eigenvectors (also known as principal components (**PCs**)), for the multinomial regression model. With the number (P) of selected **PCs** we build a multinomial regression model. In multinomial regression model we selected UD group as the reference one. The expression of the selected **PCs** (i.e. the ${\mathbf{SSF}}$ matrix reduced to the selected **PCs**, now termed ${\mathbf{SSF}}^{\prime}$) describe the effects of each particular **PC** on the quotient of the probability of a patient having the selected syndrome, by the probability of patient’s syndrome not being identified:

9 $$\begin{aligned} \ln \left( {\frac{{P_{i} \left( {PD} \right)}}{{P_{i} \left( {UD} \right)}}} \right) & = B_{0,1} + \mathop \sum \limits_{k = 1}^{P} B_{k,1} \cdot SSF_{ik}{^\prime} , \\ \ln \left( {\frac{{P_{i} \left( {MSA} \right)}}{{P_{i} \left( {UD} \right)}}} \right) & = B_{0,2} + \mathop \sum \limits_{k = 1}^{P} B_{k,2} \cdot SSF_{ik}{^\prime} , \\ \ln \left( {\frac{{P_{i} \left( {PSP} \right)}}{{P_{i} \left( {UD} \right)}}} \right) & = B_{0,3} + \mathop \sum \limits_{k = 1}^{P} B_{k,3} \cdot SSF_{ik}{^\prime} . \\ \end{aligned}$$

The model is solved using the *fitmnr* function in Matlab. It estimates coefficients, **B**, which represent weights for **PCs** for each syndrome. With the estimated coefficients **B**, the model is applied to new subjects using the *predict* function in Matlab. Output of the model consists of four predicted probabilities for each subject—one for PD, one for MSA, one for PSP, and one for the UD group. Only three predicted probabilities are independent because the sum of all four probabilities equals one. We also calculated and displayed three different topographies. They are calculated as a product of the **PC** matrix and the corresponding column of the **B** matrix. We then calculated their correlation as a Pearson correlation coefficient on a voxel level. Additional explanation of the methodology, including the equations for model predicted probabilities for each subject corresponding to either of categories, the further explanation of how coefficients **B** are determined, as well as the proofs of Eqs. 5 and 8 could be found in Appendix 1.

The model incorporated a variable number of **PCs** and a variable threshold for masking the FDG-PET images when constructing the matrix **P**. Using this algorithm, the masking threshold was varied from 15 to 45% of the FDG-PET maximum value. The number of **PCs** that were considered for the model ranged from 3 to a cumulative Variance Accounted For (VAF) not exceeding 50% [17]. The algorithm tried all different combinations of included **PC**s (e.g., for 6 selected PCs it tried all *C*(6,6) + *C*(6,5) + *C*(6,4) + *C*(6,3) = 42 combinations, where the *C*(n,k) stands for the number of *k*-combinations from a given set of *n* elements). For the final selection of **PC**s that were included in the model we searched for the optimal combination of **PCs** using the Akaike Information Criterion (AIC). The model with the lowest AIC value was deemed optimal. To assess the sensitivity of threshold selection on the algorithm performance, we added results for the second and third best models in Appendix 2.

### Model validation and diagnostic performance estimation

The diagnostic performance of the multinomial logistic regression algorithm was evaluated using FDG-PET scans from 101 validation subjects from Cohort B. ROC curves were initially computed for each patient group, comparing each group against the three other groups (i.e. two patient groups and UD). The optimal cut-off points on the ROC curves were determined using the *perfcurve* function in Matlab. This function identifies the best operating point of the model with calculation of the slope $S = \frac{{Cost\left( {P{|}N} \right) - Cost\left( {N{|}N} \right) }}{{Cost\left( {N{|}P} \right) - Cost\left( {P{|}P} \right)}}*\frac{N}{P}$, where $P = True\;Positive + False\;Negative$ and $N = True\;Negative + False\;Positive$, and finds its intersection with the ROC curve. It was assumed that the costs of false positives and false negatives were equal. Key performance metrics from ROC curves, namely the sensitivity, specificity, positive predictive value (PPV) and negative predictive value (NPV), were then calculated.

As an alternative evaluation of algorithm’s diagnostic performance, subjects were classified based on the probabilities assigned to the different groups. They were assigned to one parkinsonism group if the algorithm’s determined probability for this parkinsonism was the highest (case with no probability restriction) or above the predefined threshold (case with probability threshold for being classified in one parkinsonism group). Otherwise, they were assigned to UD group. Two probability thresholds were established (90% and 99%). With the classification based on these criteria, confusion matrices, which present true classes against predicted classes, were plotted for each threshold.

All analyses were performed using an in-house script running on Matlab 2023b, MathWorks Inc., Natick, MA.

## Results

### Model identification

The optimization of the classification model, using multinomial logistic regression and SSM/PCA, resulted in the development of a model incorporating the 1st, 2nd, 3rd, and 6th principal components (**PC**s) and FDG-PET image masking at 25% of the maximum value. The topographical representation of this optimized model yielded three distinct metabolic topographies, each highlighting metabolic characteristics that maximize the differentiation between individuals within a specific syndrome (e.g., PD) and other subjects within Cohort A.

**Fig. 3:**
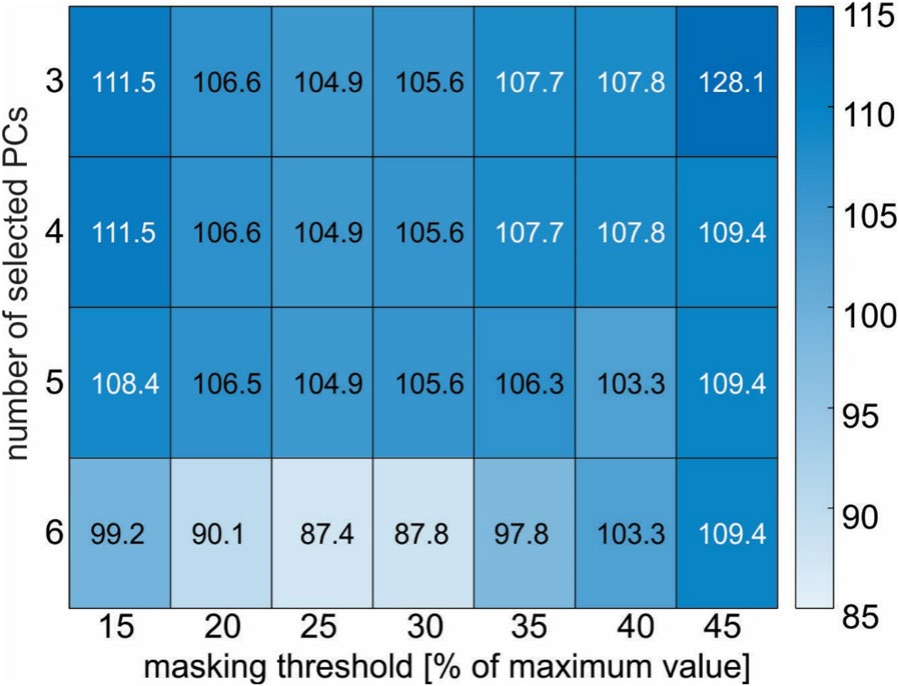
Model optimization: Akaike information criterium (AIC) values are displayed. Darker blue color means higher AIC and lighter lower AIC. The best AIC has the lightest color

During the process optimization the threshold of masking varied from 15 to 45% and the maximal number of **PC**s that are considered in the model varied from 3 to 6, ensuring that the cumulative VAF did not exceed 50% [17]. The matrix of AIC values, determined by different combination of thresholds, is presented in Fig. 3. The optimal model had an image mask threshold of 25% and a **PC** threshold of 6 (i.e., a series of logistic models were tested within the first 6 PCs). It had AIC value of 87.4 and incorporated the 1st, 2nd, 3rd, and 6th **PC**s. The image mask threshold of 30% had only 0.4% higher AIC value. Further increasing or decreasing the image mask threshold pushed the AIC value above 3% higher. However, lowering the maximal number of **PC**s included in the model result in AIC value above 18% higher, regardless of the image mask threshold. Diagnostic performance results for the second and the third best AICs are presented in Appendix 2.

**Fig. 4:**
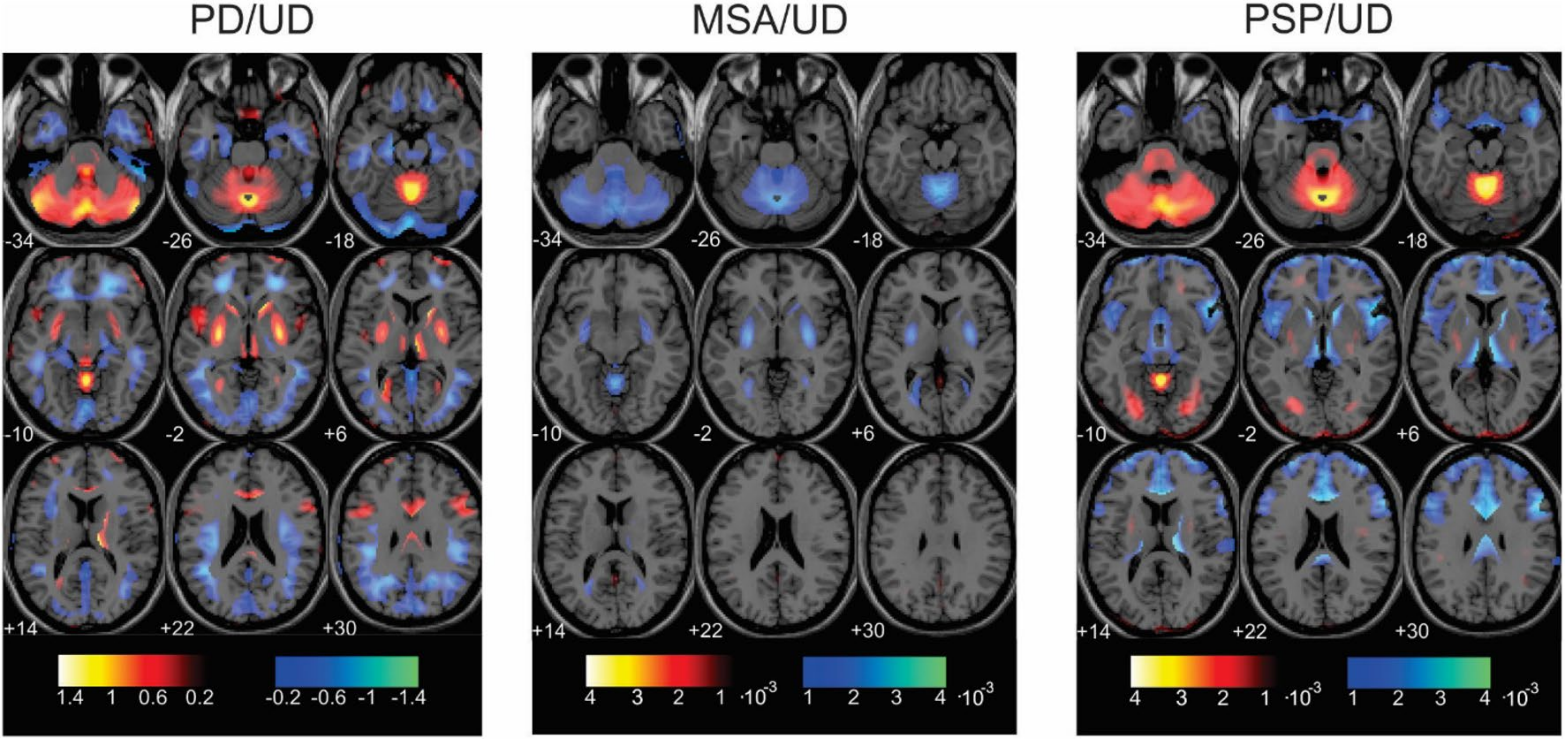
(Color should be used for this figure in print): Disease specific metabolic topographies for three different diseases: *PD/UD* Parkinson’s disease versus UD group, *MSA/UD* Multiple System Atrophy versus UD group, *PSP/UD* progressive supranuclear palsy versus UD group. The color-coded regions depict varying weights, distinguishing each syndrome from the other two based on relative metabolism

The topographic representations of the optimized model contrasting individual syndrome versus UD are displayed in Fig. 4. The PD versus UD topography (PD/UD topography) is characterized by relative hypermetabolism in the striatum and cerebellum and relative hypometabolism in the parieto-temporal and frontal cortices. The MSA versus UD topography (MSA/UD topography) is characterized by relative hypometabolism in the putamen and cerebellum. Conversely, the PSP versus UD topography (PSP/UD topography) is characterized by relative hypometabolism in the caudate nucleus and mediofrontal cortex coupled with the relative hypermetabolism in the cerebellum. We found that PD/UD and MSA/UD topographies are highly anti-correlated voxel-vise (r = − 0.81). The PD/UD and PSP/UD topographies are not correlated (r = 0.05). The MSA/UD and PSP/UD topographies are slightly anti-correlated (r = − 0.46).

### Model validation and diagnostic performance

**Fig. 5:**
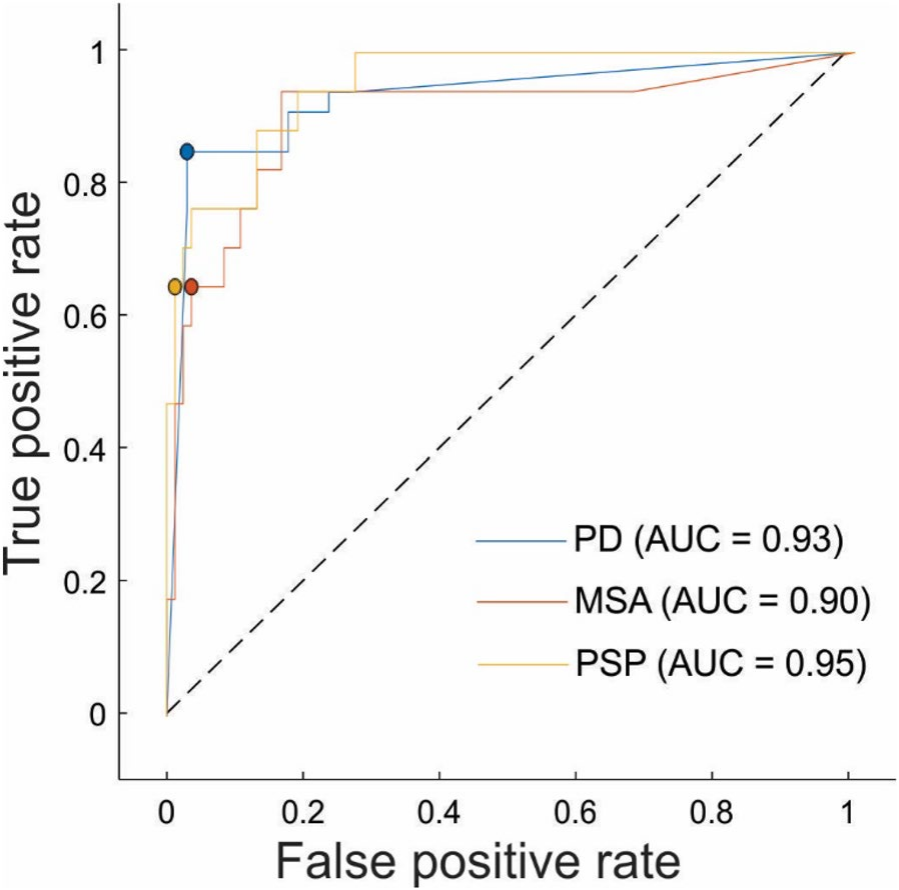
The ROC curves for Cohort B. Presentation of ROC curves and their best operating points for validation Cohort B

For all patients from the validation Cohort B, probabilities for each of the four classes (PD, MSA, PSP, UD) were calculated. ROC curves were computed for each of the three parkinsonism groups against all other groups. These ROC curves, along with their optimal operating points, are presented in Fig. 5. The AUC values were the highest for PSP (AUC = 0.95), followed by PD (AUC = 0.93) and MSA (AUC = 0.90). The model found the best operating point for PD at 0.03 false positive rate (FPR), 0.85 true positive rate (TPR); for MSA at 0.04 FPR, 0.65 TPR and for PSP at 0.01 FPR, 0.65 TPR. Calculated metrics at model’s best operating point are presented in Table 2.

**Table 2:** Performance metrics for the ROC curves for Cohort B

Group	Sensitivity (%)	Specificity (%)	PPV (%)	NPV (%)
PD	85	97	98	77
MSA	65	96	79	93
PSP	65	99	92	93

For each group sensitivity, specificity, positive predictive value (PPV) and negative predictive value (NPV) at model best operating point were calculated. Presented values demonstrate high specificity across all three groups (PD, MSA, and PSP), indicating its strong ability to correctly exclude individuals who do not have these conditions. However, the sensitivity varies, with PD showing the highest sensitivity (85%), meaning it is more effective at correctly identifying PD cases compared to MSA and PSP (both at 65%). The Positive Predictive Value (PPV) is highest for PD (98%), suggesting that a positive test result is most reliable for PD, while the Negative Predictive Value (NPV) is highest for MSA and PSP (both at 93%), indicating that a negative test result is most reliable for these conditions

**Fig. 6:**
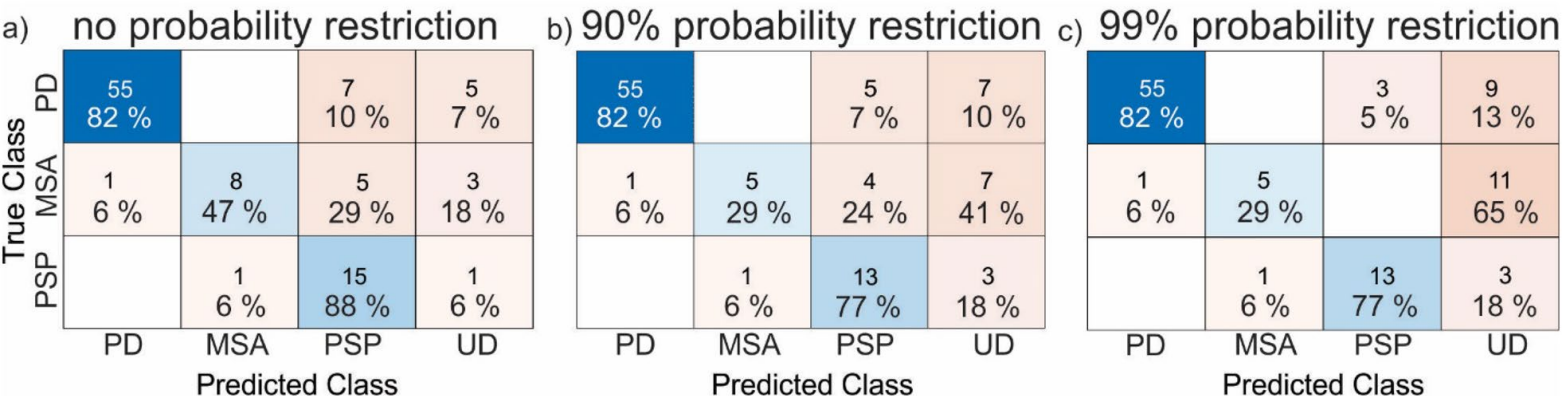
Confusion matrix. For each of the parkinsonism groups (PD, MSA, PSP), the number of validation patients and their percentage in predicted classes against true class for three classification scenarios: **a**) without probability restriction, **b**) with 90% probability threshold, and **c**) with 99% probability threshold. More intensive color present higher value. The blue color squares present properly identified subjects and orange ones’ present misclassified subjects. The value of percent’s in each row is 100%—each validation subject was included in one of predicted groups. Note that the confusion matrices are not square because all validation patients had known syndrome, so no image belonged to UD class

Confusion matrices, which present true classes against predicted classes, are presented in Fig. 6a–c. In the group with no probability threshold, 82% of PD patients, 47% of MSA patients, and 88% of PSP patients from the Cohort B were correctly predicted (Fig. 6a). In UD group there were 7% PD, 18% MSA and 6% PSP subjects. With stricter criteria for classification, when at least 90% of probability was needed to be assigned to certain syndrome group (otherwise it is assigned to the UD group), the number of correctly defined PD patients stayed the same, but an additional 2 patients were assigned to the UD group (Fig. 6b). Besides that, the number of correctly defined MSA and PSP patients were reduced to 29% and 77%, respectively. When restricting classification threshold to 99%, the number of correctly defined PD, MSA and PSP patients stayed at 82%, 29% and 77%, while only 5% of PD, 6% of MSA and 6% of PSP patients were misclassified (Fig. 6c).

## Discussion

We developed a novel, single-level, healthy-controls independent classification algorithm to differentiate among common neurodegenerative parkinsonisms using multinomial logistic regression and SSM/PCA. To do so, we first trained and optimized the algorithm on a cohort of 60 identification patients with PD, MSA, or PSP that underwent FDG-PET brain imaging for differential diagnostic purposes. Second, we presented three different metabolic topographies of the optimal model. Subsequently, we validated the model on a cohort of 101 validation patients with PD, MSA, or PSP and calculated the model diagnostic performance.

Existing algorithms for the differentiation between various parkinsonisms rely either on machine learning and direct feature extraction from FDG-PET images [31–33], or require multiple identification levels and usually the inclusion of healthy control subjects [28–30]. Our method stands out as it leverages SSM/PCA for the dimension reduction, extracting the most significant differences between all groups. It also eliminates the need for healthy control subjects in identification or validation cohorts, enabling its clinical use in the centers without local healthy control database, which could expand the utilization of these algorithms.

We used the AIC to select the optimal model, which included the 1st–3rd and 6th **PC**s and masked FDG-PET images at 25% of the maximum value. The individual principal components can represent specific factors or subtypes of the syndrome, and the eigenvalues their expression. However, this is only a possibility. Alternatively, it may be a pure mathematical construct, whereby one subtype is divided into several eigenvectors, and the specific division is defined by the imaging noise. Multinomial logistic regression process yielded three distinct discrimination metabolic topographies. They delineate the relative regional weights utilized by the algorithm to differentiate a single syndrome from the reference group, which present the average of all three syndromes (e.g., PD/UD topography). The traditional multivariate metabolic brain patterns such as PDRP [18, 40] provide characteristic metabolic changes of individual syndromes, as compared to the normal brain. Indeed, while the newly identified multinomial maps bear resemblance to disease-specific brain patterns (e.g. PDRP), they also exhibit notable differences. For example, the multinomial PD/UD topography comprises of interconnected voxels maximizing the differentiation of PD from UD group. Consequently, it encompasses not only PD-specific metabolic characteristics but also includes the information regarding MSA and PSP-specific brain metabolism.

Consistent with prior research, it is not surprising that the PD/UD topography incorporates the cerebellum, which exhibits relative hyperactivity in PDRP and hypoactivity in MSARP; putamina, which display relative hyperactivity in PDRP and hypoactivity in PSPRP; and mediofrontal regions with hypoactivity in PSPRP [18, 19, 25, 40]. Additionally, the MSA/UD topography highlights changes in the cerebellum and putamen akin to those observed in MSARP [19, 25]. The PSP/UD topography exhibits topographic similarity to the PSPRP, featuring relative hypometabolism in caudates and mediofrontal regions. However, it also incorporates the cerebellum, which is a distinguishing feature between MSARP and PSPRP.

Traditional multivariate metabolic brain patterns, such as PDRP, are valuable for understanding the disease by highlighting areas of increased or decreased metabolism. However, they might not be optimal for the mathematical formulation of differential diagnosis. For instance, in binary comparison, such as in differentiating PD and MSA, an ordinary SSM/PCA algorithm would provide direct information on how PD differs from MSA. In contrast, traditional PDRP and MSARP provide this information indirectly by comparing both syndromes against reference healthy subjects. While neither approach is inherently superior, avoiding reliance on healthy controls can be advantageous in certain scenarios.

Our multinomial logistic regression approach extends the ordinary SSM/PCA, which is typically applied to binary comparisons (e.g., PD vs. MSA) to assess multiple syndromes simultaneously without the need of healthy control subjects. The model does, however, require a reference category. To address this, we created an artificial undetermined group that resembles all three groups and serves as a reference category.

Although the disease-specific topographies of the new multinomial approach do not straightforwardly depict disease-specific brain networks (such as the classical multivariate SSM/PCA-derived patterns), such representation provides crucial insights into the distinguishing features. These features are typically unavailable in conventional machine learning approaches. The presented topographies highlight the regions that physicians primarily focus on when visually evaluating FDG-PET brain scans in the context of neurodegenerative parkinsonisms [8].

Our algorithm rarely misclassified PD and MSA cases; there was only one case in which MSA was misclassified as PD, while the opposite never happened. This is likely due to the high anti-correlation between the PD/UD and MSA/UD topographies. The PD/UD topography is quite distinct, so it is unlikely for PD patient to be misclassified as MSA. MSA and PSP are somewhat more frequently misclassified, especially when there is a low probability threshold for the categorization into one of the three syndromes. This is probably because their topographies are slightly anti-correlated and both have a small magnitude (are less distinct). PD is sometimes misclassified as PSP, as their topographies are uncorrelated. Interestingly, the opposite never happened—the PSP is never misclassified as PD. On the other hand, one MSA case, whose topography is anti-correlated with the PD/UD topography was "reliably" classified as PD.

The multinomial algorithm demonstrates comparable results with prior metabolism-based differential diagnostic algorithms in terms of AUC values, sensitivity, specificity, PPV, NPV at the model's best operating point, and the number of correctly predicted subjects’ diagnosis. Tripathi et al. [29] reported 2–4% higher AUC values for all identification groups, compared to ours.

We compared metrics from ROC curves of our validation results with three two-level studies (Tang et al. [28], Tripathi et al. [29] and Rus et al. [30]) and with two machine learning algorithms (Zhao et al. [33] and Garraux et al. [32]). With our selected cut off value our sensitivity results for PD were in the middle of others, but for MSA and PSP they were worse than all others reported; for PD sensitivity was 85%, while in other studies were between 50 and 98%, for MSA our sensitivity was 65%, and in other studies were between 66 and 100% and for PSP our sensitivity was 65%, and in other studies were between 67 and 100%. Specificity of our algorithm was above the best in comparison with others (for PD our specificity was 97%, others were between 82 and 100%, for MSA our was 96%, others were between 82 and 100% and for PSP our was 99%, and others were between 77 and 100%). In PPV and NPV metrics our algorithm was somewhere in the middle of others. The PPV was 98% for PD cases in our study while others reported PPV between 82 and 100%, for MSA our was 79%, others were between 67 and 100% and for PSP our was 92%, and others were between 75 and 100%. The NPV was 77% for our PD cases and others were between 50 and 97%, for MSA our was 93%, others were between 66 and 100% and for PSP our was 93%, others were between 50 and 100%.

In confusion matrices Garraux et al. [32] and Mudali et al. [31] reported up to 8% higher correct predictions for PD cases. For the MSA cases, our results were in the middle of theirs (their results were 2% lower and 24% higher) and for the PSP their results were up to 41% lower than ours.

In light of these findings, it is evident that the performance metrics of our algorithm is similar to those reported in previous studies. This novel approach, while being simpler and more flexible, offers a comparable accuracy and reliability in diagnosing PD, MSA, and PSP. By adjusting prediction thresholds, we can influence the number of subjects assigned to the UD group. Higher thresholds result in a fewer correctly predicted subjects but also fewer misclassifications. In a clinical setting, assignment to the UD group could alert clinician to run additional tests for those subjects. Our algorithm outperforms others in certain metrics (i.e. specificity) but falls short in others (i.e. sensitivity). As the algorithm is primarily used as a confirmatory test for clinicians, high specificity is most important; especially as such algorithms are indispensable in recruiting patients in clinical trials where high specificity is crucial [24, 41] and reliable biomarkers are missing.

Within the optimization process of the multinomial logistic regression algorithm we have shown that the exact selection of the masking threshold for FDG-PET image is not critical for the successful classification. We masked FDG-PET images at 25% of the maximum value, but masking at 20% or 30% of the maximal value produced at most 0.06 difference in AUC results (Appendix 2). It should also be noted that the optimal FDG-PET image masking threshold depends on the preprocessing steps for FDG-PET images, like the smoothing filter, and the intrinsic resolution of the FDG-PET images, which depend on the scanner used and image reconstruction parameters. In our prior study on Alzheimer's disease-related patterns, we have demonstrated that the diagnostic accuracy remains robust regardless of the resolution of FDG-PET images [37] and the results of this current study are in line with this.

An important advantage of our algorithm against the two level algorithm based on SSM/PCA [28–30] is its ability to distinguish between all types of parkinsonisms simultaneously and with no need of FDG-PET scans from healthy controls. It can also easily accommodate additional new patient groups. A key advantage of our algorithm against the algorithms based on machine learning [31–33] is a known and fully understood mechanism of decision making in the classification process.

The implementation of our algorithm is set to have equal number of PD, MSA and PSP patients in the identification cohort, so all three syndromes are equally represented. Therefore, the outcome of our algorithm is not affected by the incidence of particular syndrome in the population. Knowing that PD is far more common than MSA and PSP, the outcome of our algorithm could be supplemented by the population incidence using the Bayesian statistics. This way one could calculate the best estimate of the probability for each syndrome. However, parkinsonian patients are not routinely referred to a rather costly FDG-PET imaging for the differential diagnosis. In a subpopulation of parkinsonian patients that undergo FDG-PET imaging for the differential diagnostic purposes, the incidence of PD is not much different from the incidence of MSA or PSP. This is evident from the sample size for each syndrome, which is far more uniform than one would expect based on the population incidence. Therefore, the outcome of our algorithm could be supplemented with the Bayesian statistics only if the incidence of each parkinsonism is known for the population that is referred to the FDG-PET imaging for the differential diagnostic purposes. However, incorporating that into the model would be a substantial extension of our approach. In addition to that, such an extension is plausible for many existing differential diagnostic algorithms. Therefore, we leave that idea for possible future work.

Our study does have certain limitations. This is a single center study and the number of cases could be increased to provide more robust and reliable results [37]. Furthermore, it exclusively distinguishes among the three most prevalent neurodegenerative parkinsonian syndromes, while for broader clinical practice additional groups of patients should be added. Validation *of our results* in multiple centers is crucial to test the algorithm's performance across diverse patient populations and clinical practices, thereby confirming its applicability in various real-world settings. Moreover, using different FDG-PET scanners is essential to account for potential variability in imaging results due to differences in scanner calibration, image reconstruction algorithms, and acquisition parameters. This would help ensure that the algorithm remains reliable and reproducible regardless of the technical environment, ultimately enhancing its clinical utility and acceptance. Testing the algorithm on patients with early stage of the disease would also provide valuable insight into the algorithm performance. Another limitation of our study is the absence of histologically confirmed diagnosis. Although the movement disorder specialist confirmed the diagnosis at least one year after the imaging, an additional imaging of presynaptic dopaminergic integrity may further increase the diagnostic accuracy. Additional studies would be warranted to validate the algorithm on images from different scanners or centers to ensure its robustness and generalizability.

## Conclusion

This novel classification algorithm, based on multinomial logistic regression and SSM/PCA, demonstrates promising results in classifying and distinguishing between various types of parkinsonisms. The algorithm exhibits robust performance metrics and offers advantages over existing methods. Notably, it can distinguish between multiple types of parkinsonisms simultaneously with no need of healthy control images. Additionally, it can easily accommodate new patient groups. It also allows a clear insight into the decision making process. Future work shall focus on incorporating more patient groups and larger identification groups to further validate its usability for the differential diagnosis of parkinsonisms in clinical settings and research.

## Supplementary Information

Additional file 1.

Additional file 2.

Additional file 3.

## Figures and Tables

**Fig. 7:**
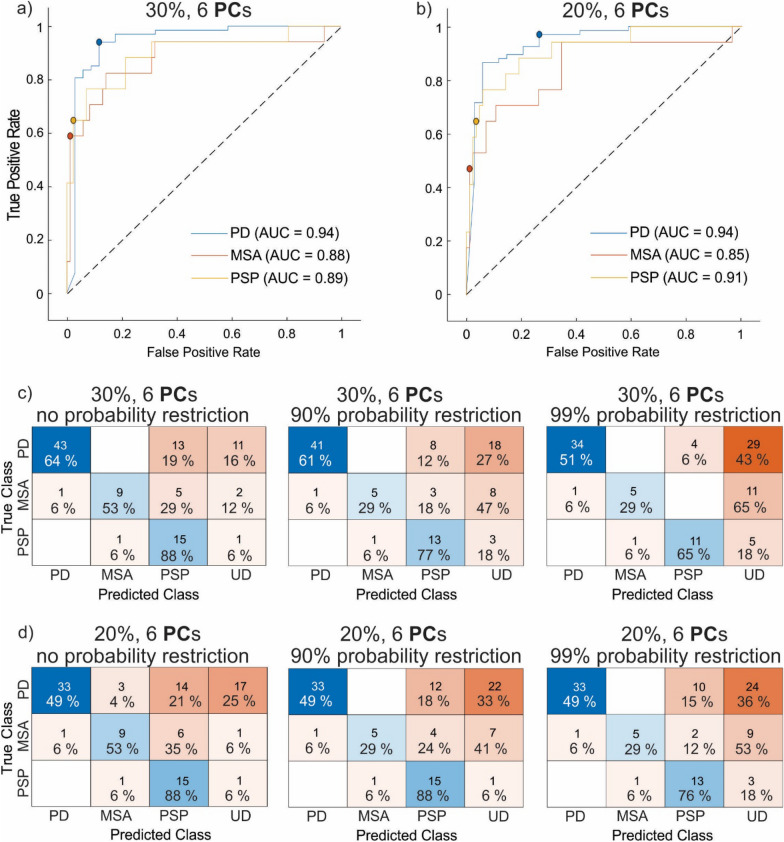
**a**) The ROC curves for Cohort B for masking threshold of 30% and 6 **PC**s. **b**) The ROC curves for Cohort B for masking threshold of 20% and 6 **PC**s. **c**)Confusion matrices for masking threshold of 30% and 6 **PC**s. **d**) Confusion matrices for masking threshold of 20% and 6 **PC**s. For each of the syndrome groups (PD, MSA, PSP), the number of validation patients and their percentage in predicted classes are presented against true class for three classification scenarios: without probability restriction, with 90% probability threshold, and with 99% probability threshold. More intensive color in squares present higher value. The blue color squares present properly identified subjects and orange ones’ present misclassified subjects. The value of percent’s in each row is 100%—each validation subject was included in one of predicted groups. Note that the confusion matrices are not square because all validation patients had known syndrome, so no image belonged to UD class

## Data Availability

Data and code from the study is available from the corresponding author on reasonable request, after clearance from Research Ethics Committee. PD, MSA in PSP topographies are available in Supplementary material.

## Appendix 1

Equation 5 can be proved by multiplying the Eq. 4 with the ${\mathbf{SRP}}^{{\mathbf{T}}}$ from the left:

10 $${\mathbf{SRP}}^{{\mathbf{T}}} \cdot {\mathbf{S}}_{{{\mathbf{sub}}}} \cdot {\mathbf{E}} = {\mathbf{SRP}}^{{\mathbf{T}}} \cdot {\mathbf{E}} \cdot {\Lambda },$$

11 $${\mathbf{SRP}}^{{\mathbf{T}}} \cdot {\mathbf{SRP}} \cdot {\mathbf{SRP}}^{{\mathbf{T}}} \cdot {\mathbf{E}} = {\mathbf{S}}_{{{\mathbf{vox}}}} \cdot {\mathbf{SRP}}^{{\mathbf{T}}} \cdot {\mathbf{E}} = {\mathbf{SRP}}^{{\mathbf{T}}} \cdot {\mathbf{E}} \cdot {\Lambda }.$$

Equation 8 can be proved by multiplying the Eq. 7 with ${\mathbf{GIS}}$ the from the right:

12 $$\begin{aligned} {\mathbf{SRP}} \cdot {\mathbf{GIS}} & = {\mathbf{SSF}} \cdot {\mathbf{GIS}}^{{\varvec{T}}} \cdot {\mathbf{GIS}} = {\mathbf{SSF}} \cdot \left( {{\mathbf{SRP}}^{{\varvec{T}}} \cdot {\mathbf{E}} \cdot {\Lambda }^{{ - \frac{1}{2}}} } \right)^{{\varvec{T}}} \cdot {\mathbf{SRP}}^{{\varvec{T}}} \cdot{\mathbf{E}}\cdot{\Lambda }^{{ - \frac{1}{2}}} \\ & = {\mathbf{SSF}} \cdot {\Lambda }^{{ - \frac{1}{2}}} \cdot {\mathbf{E}}^{{\varvec{T}}} \cdot {\mathbf{SRP}} \cdot {\mathbf{SRP}}^{{\varvec{T}}} \cdot{\mathbf{E}}\cdot{\Lambda }^{{ - \frac{1}{2}}} = {\mathbf{SSF}} \cdot {\Lambda }^{{ - \frac{1}{2}}} \cdot {\mathbf{E}}^{{\varvec{T}}} \cdot {\mathbf{S}}_{{{\mathbf{sub}}}} \cdot{\mathbf{E}}\cdot{\Lambda }^{{ - \frac{1}{2}}} \\ & = {\mathbf{SSF}} \cdot {\Lambda }^{{ - \frac{1}{2}}} \cdot {\mathbf{E}}^{{\varvec{T}}} \cdot {\mathbf{E}} \cdot {\Lambda }\cdot{\Lambda }^{{ - \frac{1}{2}}} = {\mathbf{SSF}}, \\ \end{aligned}$$

13 $${\mathbf{SSF}} = {\mathbf{SRP}} \cdot {\mathbf{GIS}} = {\mathbf{SRP}} \cdot {\mathbf{SRP}}^{{\varvec{T}}} \cdot{\mathbf{E}}\cdot{\Lambda }^{{ - \frac{1}{2}}} = {\mathbf{E}} \cdot {\Lambda }\cdot{\Lambda }^{{ - \frac{1}{2}}} = {\mathbf{E}} \cdot {\Lambda }^{\frac{1}{2}} .$$

The **B** coefficients are determined by the multinomial regression analysis of the observed data. For each observation, the outcome is a specific category that subject belongs to. During the fitting process, the model estimates the log-odds of each category relative to the reference category. These log-odds are calculated according to the Eq. 9., using the current **B** coefficients and the following formulas:

14 $$\begin{aligned} P_{i} \left( {UD} \right) & = \frac{1}{{1 + \mathop \sum \nolimits_{j = 1}^{3} {\text{Exp}}\left[ {B_{0,j} + \mathop \sum \nolimits_{k = 1}^{P} B_{k,j} \cdot SSF_{ik}{^\prime} } \right]}} \\ P_{i} \left( {PD} \right) & = \frac{{{\text{Exp}}\left[ {B_{0,1} + \mathop \sum \nolimits_{k = 1}^{P} B_{k,1} \cdot SSF_{ik}{^\prime} } \right]}}{{1 + \mathop \sum \nolimits_{j = 1}^{3} {\text{Exp}}\left[ {B_{0,j} + \mathop \sum \nolimits_{k = 1}^{P} B_{k,j} \cdot SSF_{ik}{^\prime} } \right]}} \\ P_{i} \left( {MSA} \right) & = \frac{{{\text{Exp}}\left[ {B_{0,2} + \mathop \sum \nolimits_{k = 1}^{P} B_{k,2} \cdot SSF_{ik}{^\prime} } \right]}}{{1 + \mathop \sum \nolimits_{j = 1}^{3} {\text{Exp}}\left[ {B_{0,j} + \mathop \sum \nolimits_{k = 1}^{P} B_{k,j} \cdot SSF_{ik}{^\prime} } \right]}} \\ P_{i} \left( {PSP} \right) & = \frac{{{\text{Exp}}\left[ {B_{0,3} + \mathop \sum \nolimits_{k = 1}^{P} B_{k,3} \cdot SSF_{ik}{^\prime} } \right]}}{{1 + \mathop \sum \nolimits_{j = 1}^{3} {\text{Exp}}\left[ {B_{0,j} + \mathop \sum \nolimits_{k = 1}^{P} B_{k,j} \cdot SSF_{ik}{^\prime} } \right]}} \\ \end{aligned}$$

In each iteration step, the **B** coefficients are updated until the predicted probabilities according to model fits the observed probability. In this way the **B** coefficients are determined.

## Appendix 2

Model with mask threshold at 30% and **PC** threshold 6 had slightly worse AUC results on validation group (1% higher for PD, 2% lower for MSA, 6% lower for PSP; Fig. 7a). With confusion matrix it could be seen that a bit less PD subjects were properly identified (18%), and few more MSA subjects were properly identified (6%). As with the optimal setting, the higher probability restriction implies less subjects in proper group, but also less misclassified and more in UD group (Fig. 7c).

Model with mask threshold 20% and PC threshold 6 had also slightly worse AUC results on validation group for all three syndromes (1% higher for PD, 5% lower for MSA and 4% lower for PSP; Fig. 7b). From the confusion matrix it could be seen that the number of properly identified PD was worse for 33%, and few more MSA subjects were properly identified (6%). With higher probability restrictions trends are similar (Fig. 7d).
